# Comparative Analysis of Polyphenolic Acids from Various *Zea mays* Parts in Ultrasound-Assisted Extraction

**DOI:** 10.3390/foods14091458

**Published:** 2025-04-23

**Authors:** David Řepka, Lubomír Lapčík

**Affiliations:** 1 Department of Physical Chemistry, Faculty of Science, Palacky University, 17. Listopadu 12, 771 46 Olomouc, Czech Republic; david.repka@upol.cz; 2 Department of Foodstuff Technology, Faculty of Technology, Tomas Bata University in Zlin, Nam. T.G. Masaryka 275, 762 72 Zlin, Czech Republic

**Keywords:** antioxidants, polyphenols, therapeutic applications, ultrasound extraction, *Zea mays*

## Abstract

In this study, we compared different parameters in the ultrasound-assisted extraction of polyphenolic acids from seven parts of *Zea mays* (kernels, leaves, stems, corn silks, roots, the whole plant, and the whole fermented plant) to identify its richest natural sources. Additionally, the correlation between extraction parameters and polyphenol yield was investigated. The extraction was performed using ultrasound at varying powers (480 or 240 W) and frequencies (80 or 37 kHz). Total phenolic content (TPC) was determined using the Folin–Ciocalteu assay, while radical scavenging activity (RSA) was assessed via the DPPH assay. The TPC values ranged from 0.69 ± 0.00008 mg GAE/g to 4.07 ± 0.0004 mg GAE/g in corn. RSA analysis revealed the highest scavenging activity in corn silk (80.06% ± 1.01) and the lowest in kernels (2.77% ± 0.90). High-performance liquid chromatography identified up to 22 different phenolic acids per sample, with the 5 most abundant being chlorogenic acid, protocatechuic acid ethyl ester, quercetin, sinapic acid, and *trans*-cinnamic acid. The study found small effects of power and frequency on the extraction efficiency. This suggests a practical advantage for industrial-scale applications, as using 240 W instead of 480 W under the same conditions can reduce energy consumption without compromising yield.

## 1. Introduction

The global production of corn for the 2022/2023 season was around 1.16 billion metric tons [1]. This plant has a wide range of uses, i.e., a human food source, feed for livestock, biofuel, or in industry [2]. *Zea mays* is one of the most important plant-based food sources in the world. In populations where corn is one of the main foodstuffs, the dependence on corn can lead to the development of some health issues, such as pellagra, a disease caused by niacin deficiency, as corn lacks sufficient amounts of this essential nutrient [2,3]. Niacin plays a crucial role in cellular metabolism and the body’s defense against oxidative damage [4].

For many years, antioxidants have been known for their ability to fight against oxidative stress, which occurs when the balance between reactive oxygen species (ROS) and antioxidants is disrupted. Oxidative stress is not only linked to major diseases, such as cancer, diabetes, skin damage, cardiovascular diseases, Alzheimer’s, Parkinson’s, and chronic inflammation, but also plays a role in nutrient deficiency-related disorders such as pellagra [5,6,7,8,9,10]. A study by Tang et al. found that niacin deficiency causes oxidative damage to proteins and DNA in the bone marrow cells of rats [6].

**Figure 1:**
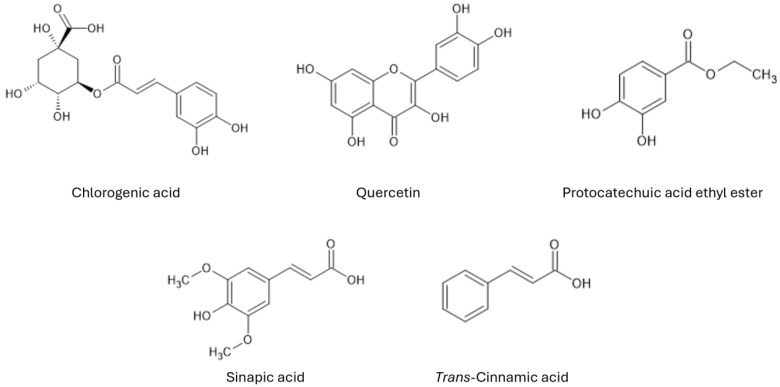
Structures of the five polyphenolic acids found in the highest concentrations in various *Zea mays* parts.

Polyphenols, shown in Figure 1, are a group of naturally occurring substances that contain mainly antioxidant properties but can also exhibit pro-oxidant effects [11]. With more than 8000 identified polyphenols, these secondary metabolites can be found in various plants, fruits, and vegetables, such as broccoli, onions, blueberries, grapes, apples, tea, coffee, and many more [12,13,14,15,16]. The concentration of polyphenols in the plant matrix depends on numerous factors, including plant species; environmental conditions such as altitude, temperature, and light or maturity level; processing; and even storage [17,18,19]. The concentration of polyphenols can range from a few dozen milligrams per 100 g to more than 15,000 mg per 100 g. For example, common foods such as broccoli contain 45 mg, filtered coffee contains 214 mg, and cloves have one of the highest contents, with 15,188 mg per 100 g [20]. Polyphenols are also present in *Zea mays*. For example, in a review carried out by Sánchez-Nuño et al., the total phenolic content of kernels from different maize varieties ranged from 34.7 to 61 mg gallic acid equivalent (GAE) per 100 g, as determined via total phenolic content (TPC) assays [21]. In another study by Feregrino-Pérez et al., an analysis of five different cobs was conducted, yielding a range of total phenols from 202 ± 3.53 to 5482.5 ± 34.86 mg GAE per 100 g [22].

Polyphenols show promising health benefits in different therapeutic applications. Apart from their significant antioxidant activity, they can also exhibit anti-inflammatory, antidiabetic, anticancer, antibacterial, antiviral, and antifungal properties, to name a few [23,24].

Antioxidants in an enzymatic and a non-enzymatic form behave as a defense against oxidative stress by neutralizing free radicals, thus protecting cells from damage [25,26]. Recent studies have shown that combining multiple antioxidants can be more effective than using just one type, forming a so-called synergy [27,28,29,30].

**Figure 2:**
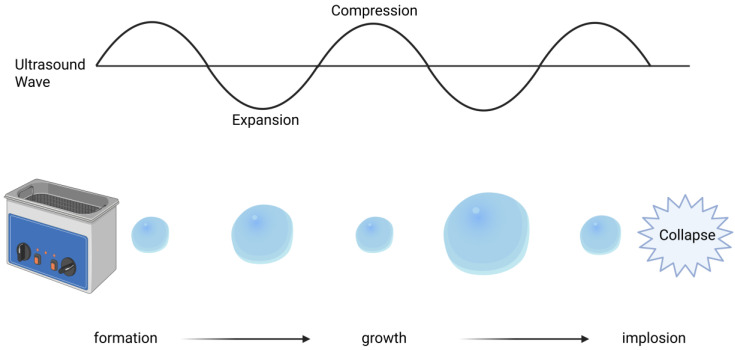
Graphical representation of effect of ultrasound on solvent. Created in BioRender. Řepka, D. (2025) https://BioRender.com/gzze4ep (accessed on 14 April 2025).

In this article, the extract of *Zea mays* parts was used without separating any compounds. For our study, ultrasound was used, which can be described as sound waves with a frequency of 20 kHz or higher [31,32]. Ultrasound-assisted extraction (UAE) is based on energy transfer to the solvent, which leads to pressure changes. These changes generate so-called cavitation bubbles. The creation of cavitation bubbles consists of formation, growth and then collapse, as can be seen in Figure 2 [31,33,34,35]. The collapse of medium bubbles produces high temperature and pressure energy, resulting in shock waves. Generated shock waves contribute to structural changes on the surface of the plant material. Because of these changes, the solvent can penetrate the plant matrix and extract the substance [34,36,37,38]. The benefits of UAE include its environmental sustainability, ease of use, and cost-effectiveness [39,40,41,42]. In a study by Sawangwong et al., UAE was applied to corn silk samples alongside other conventional methods, demonstrating that UAE is the preferred technique, as it yields a greater amount of polyphenols [43]. In another study on purple corn, Boateng et al. similarly found that UAE extracts exhibited increased antioxidant properties and required less extraction time compared with the results for microwave-assisted extraction [44]. Studies on polyphenolic extraction from *Zea mays* have mostly focused on a few parts of the plant, leaving others unexplored. The purpose of this study was to evaluate the polyphenolic content and radical scavenging capacity of different parts of *Zea mays* in order to compare extracts obtained from the whole plant and fermented whole plant and to assess the influence of various ultrasonic-assisted extraction parameters on extraction efficiency and potential energy optimization.

## 2. Materials and Methods

### 2.1. Chemicals

2,2-Diphenyl-1-picrylhydrazyl (DPPH), gallic acid, quercetin (≥95% HPLC), and L-ascorbic acid (99%) were purchased from Sigma-Aldrich (St. Louis, MO, USA). Ethanol (96% guaranteed reagent grade) was purchased from Lach-Ner (Neratovice, Czech Republic), sodium carbonate was purchased from Penta Chemicals (Prague, Czech Republic), and Folin–Ciocalteu’s phenol reagent (FCR) was obtained from Merck (Darmstadt, Germany).

### 2.2. Sample Preparation

**Figure 3:**
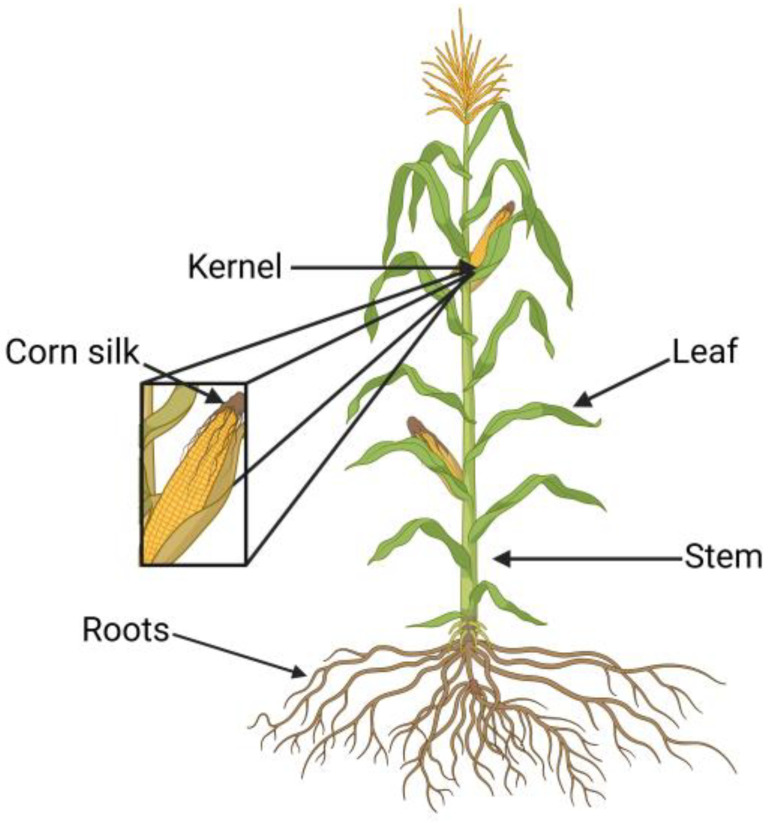
*Zea mays* plant with parts used for extraction. Created in BioRender. Řepka, D. (2025) https://BioRender.com/jpk2icn (accessed on 14 April 2025).

Ten randomly selected *Zea mays* plants were collected from different locations in a field in the Olomouc Region of the Czech Republic. They were harvested during their R3 stage of growth and then air-dried. The maize plant was divided into seven different parts—kernel (F), stem (S), leaf (L), corn silk (CS), root (R), the whole plant (KCM), and the fermented whole plant (KCF)—as shown in Figure 3.

Small portions of different dried parts were collected and crushed using a Sencor SHB 4310 device (Sencor, Říčany, Czech Republic). The remaining plants, which consisted of all parts (F, S, R, CS, and L), were crushed in the same manner. A fermented whole plant sample was collected from a silage pit. The plants from a field were crushed as a whole and left in a silage pit for one year to ferment. The whole plant and fermented whole plant were selected to act as controls.

### 2.3. Ultrasound-Assisted Extraction

The prepared samples were subjected to ultrasound for 20 min at a 1:10 S/L ratio, with 70% ethanol as the solvent. The extraction was performed using four different parameters for each sample. The specific settings are given in Table 1.

Ultrasonic treatment was carried out using a Fisherbrand FB-11201 device (Thermo Fischer Scientific, Waltham, MA, USA), operating in pulse mode. The specific dynamics of the pulse mode are proprietary to the manufacturer and not publicly disclosed. The device delivers a nominal ultrasonic power of 400 W, which can reach up to 480 W in pulse mode. For the experiments, two power levels were used: 50% and 100% of the maximum pulse output, approximately corresponding to 240 W and 480 W, respectively. The temperature was not a primary variable in this study. However, it was monitored throughout the extraction process, ranging from 22 °C at the start of extraction to 51 °C at the end of the extraction procedure. After extraction, the samples were filtered through filter paper and then centrifuged. The samples were kept at 4 °C until further use.

### 2.4. UV–Vis Analysis

Each sample underwent UV–Vis analysis. For this, an Analytic Jena AG Specord S 600 device (Jena, Germany) was used, covering a range of 240 to 400 nm. The blank used was 70% ethanol.

### 2.5. Attenuated Total Reflectance Analysis

An attenuated total reflectance (ATR) analysis of the ethanolic extracts was performed using a Nicolet iS50 FTIR spectrometer (Thermo Fischer Scientific, Waltham, MA, USA). The settings for the measurement were set to 32 scans, with a resolution of 4 cm^−1^ from 4000 to 400 cm^−1^.

### 2.6. Total Phenolic Content

Total phenolic content was determined using the Folin–Ciocalteu redox assay. The procedure reported by Molole et al. [45] was used, with slight modification. Prior to the experiment, the FCR was diluted in a 1:10 ratio with distilled water. Sodium carbonate (7.5% *w*/*v*) and gallic acid were prepared as a standard. The concentrations of gallic acid used were as follows: 10, 20, 30, 40, 60, 80, and 100 ppm. Following the procedure, 250 μL of gallic acid or sample extract was transferred into a test tube together with 1 mL of FCR. After 5 min of incubation, 2 mL of Na_2_CO_3_ was added and vortexed. Samples were incubated in the dark for 30 min, and UV–Vis spectra were measured at 760 nm. The total polyphenol content was calculated as the gallic acid equivalent per gram of dry sample using the following equation:

(1) $$c=c_{1}\times \frac{V}{m}$$

where *c* is the total phenolic content in mg/g as the gallic acid equivalent (GAE), *c*_1_ is the concentration calculated from the calibration curve in mg/mL, *V* stands for the volume of the extract in mL, and *m* is the mass of the dry plant sample in g. All samples were measured in triplicate.

### 2.7. Radical Scavenging Activity

A DPPH solution of 6 × 10^−6^ mol/L was used to determine the radical scavenging activity. A fresh solution of DPPH was prepared prior to the experiment. For the test, 2.9 mL of DPPH and 10 µL of the sample extract were mixed and incubated for 30 min in the dark, and absorption at 517 nm was measured. Ascorbic acid as a standard and a control sample were prepared and analyzed as previously stated. Radical scavenging activity was calculated based on the following equation:

(2) $$\%\;inhibition\;of\;DPPH\;radical=\frac{A_{c}-A_{s}}{A_{c}}\times 100$$

where *A_c_* is the absorbance of the control, and *A_s_* is the absorption of the sample. Based on the standard calibration curve, the ascorbic acid equivalent per gram of dry sample was also calculated. All samples were measured in triplicate.

### 2.8. HPLC Analysis

High-performance liquid chromatography was performed using Thermo Scientific^TM^ Dionex^TM^ UltiMate^TM^ HPLC system (Thermo Fischer Scientific, USA). All samples were tested in duplicate. The samples for analysis were filtered using a 0.2 μm filter membrane. The chromatographic column used was the Phenomenex Kinetex C18 (150 mm × 4.6 mm; 5 μm). Mobile phase A comprised 99:1 water/99.8% glacial acetic acid; mobile phase B was made up of 67:32:1 water/acetonitrile/99.8% glacial acetic acid. The detection wavelength was 275 nm; the column temperature was 25 °C; the injection volume was 50 μL; the flow rate was 0.700 mL/min at 54 bar; an external standard method was used; and the processing time was 45 min. The mobile phase gradient elution was as follows: 0–10 min, 10–20% B; 10–16 min, 20–40% B; 16–20 min, 40–50% B; 20–25 min, 50–70% B; 25–30 min, 70% B; 30–40 min, 70–10% B; 40–45 min, 10% B [46].

Complete graphs showing all the polyphenolic acids and their concentrations in each sample can be found in the Supplementary Materials (Figures S1–S7).

### 2.9. Statistical Analysis

A two-way repeated-measures analysis of variance (ANOVA) was conducted to identify significant differences in the used extraction settings, with statistical significance defined as a *p*-value < 0.05. Tukey’s post hoc test was carried out for every ANOVA test with a *p*-value of <0.05.

## 3. Results and Discussion

### Quantitative and Qualitative Analysis of Polyphenols in Maize Samples

**Figure 4:**
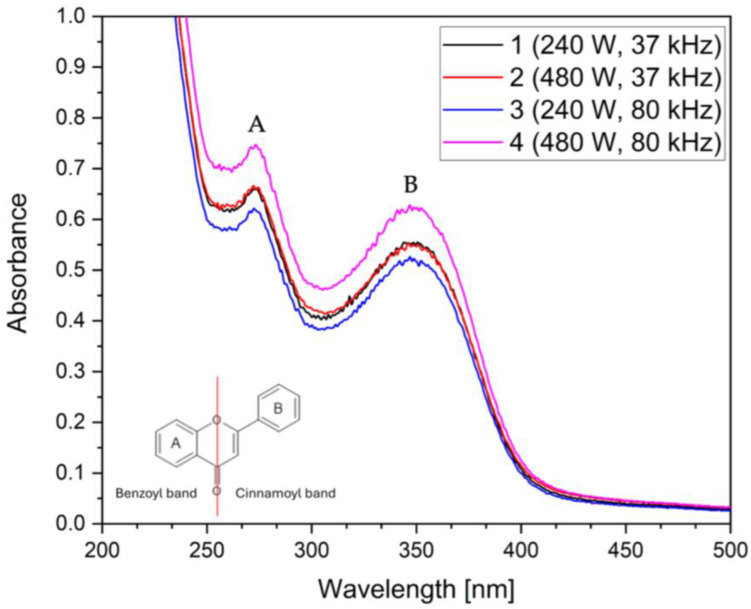
UV–Vis spectra of corn silk (CS) samples 1–4.

The UV–Vis analysis of the extracts demonstrated two main peaks, at approximately 270 and 320–350 nm. The first peak is correlated with the benzoyl group and the second with the cinnamoyl group [47,48,49]. In Figure 4, the corn silk sample shows distinct groups, such as those found in the structure of flavonoids, a subgroup of polyphenols. The corn silk sample showed the greatest peak separation, while the leaves showed the highest absorbance. All other samples demonstrated varying degrees of absorbance and peak separation, but the presence of both benzoyl and cinnamoyl groups was evident. The corresponding figures can be found in the Supplementary Materials, Figures S8–S21.

**Figure 5:**
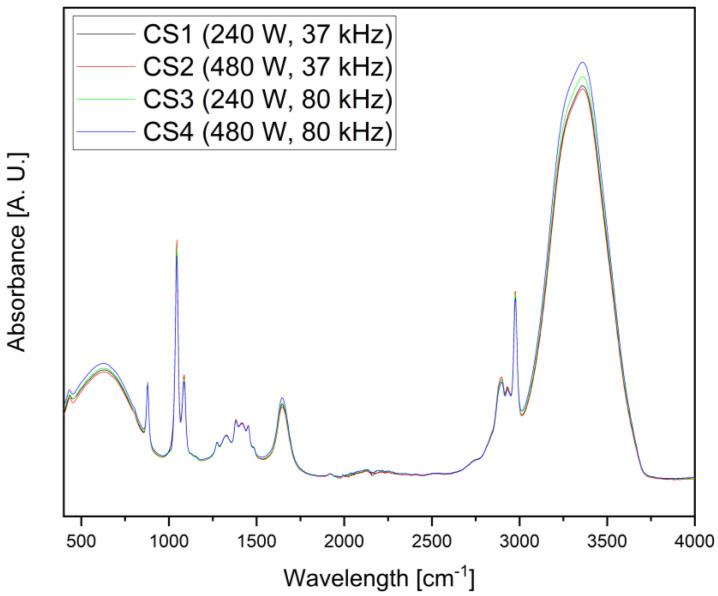
ATR spectra of ethanolic extracts for corn silk samples 1–4.

IR spectra were analyzed to confirm the presence of polyphenols. Figure 5 shows the IR spectra of ethanolic extracts obtained by ATR. According to the literature, in the range of 900–600 cm^−1^, bands correspond to C–H vibrations in aromatic rings [50,51,52] or C–C stretching vibrations in ethanol [53]. The major bands around 1085 to 1044 cm^−1^ can correlate to C–H ring vibrations or C–O stretching and C–OH bending in alcohols, ethers, esters and carboxylic acid, as well as to the ketone group, in the case of the second band [50,52,53,54]. Multiple bands in the area ranging from approximately 1430 to 1260 cm^−1^ could be related to the CH_3_, CH_2_, and bending vibration of C–H, as well as the stretching vibration in the aromatic rings [53]. The band at 1646 cm^−1^ is attributed to the C=C stretching vibration in aromatic rings of the polyphenols, as well as the C=O stretching vibration [50,52,53]. Bands in the range from 3000 to 2900 cm^−1^ represent the stretching of the CH_3_ and CH_2_ groups, while the peak at 2975 cm^−1^ represents the C–H in ethanol. The final peak at 3359 cm^−1^ is believed to correspond to the OH group [53]. In Figure 5, CS refers to corn silk; the numbers correspond to the extraction parameters listed in Table 1.

**Figure 6:**
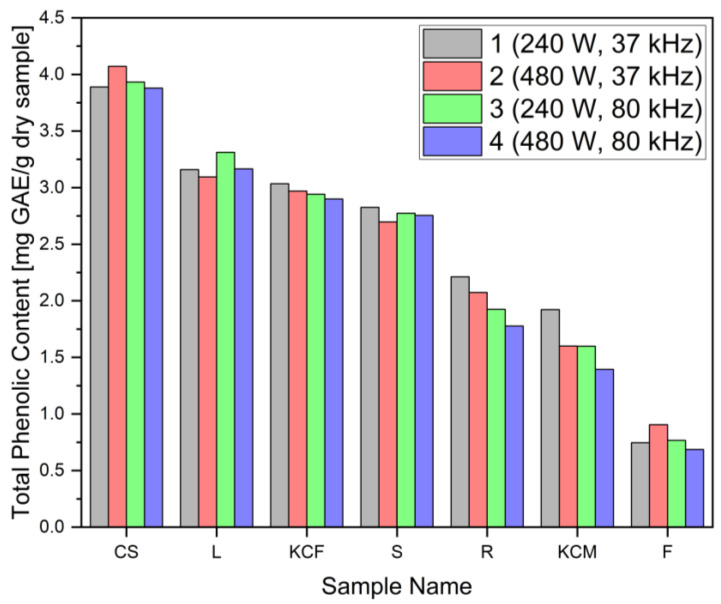
Total phenolic content of seven maize parts calculated as gallic acid equivalent per g of dry sample.

The total phenolic content, as determined by the Folin–Ciocalteu redox assay, showed the highest concentration of phenolic substances in corn silk sample 2 (4.07 mg GAE/g). On the other hand, the lowest concentration was found in kernel sample 4 (0.69 mg GAE/g). Leaves, stems, and fermented whole plants showed similar results for total phenolic content, ranging from approximately 2.8 to 3.1 mg GAE/g. Samples of the roots exhibited values from 1.78 to 2.21 mg GAE/g. The last sample, whole plant (KCM), exhibited values from 1.4 to 1.92 mg GAE/g. The results can be seen in Figure 6. The standard deviation was calculated from the triplicate measurement and is given in the graph, but due to very small deviations, it is hardly visible. Statistical analysis showed no significant correlation between power and frequency for obtaining total phenolic content, apart from the root (R) sample. In this sample, the combination of 240 W with 37 kHz frequency was observed as the best option for achieving a higher concentration of polyphenols. In the study by Suriano et al., four different genotypes of maize kernels were investigated by TPC assays [55]. It was found that TPC ranged from 1359 ± 34.4 to 4047 ± 268.2 μg/g of catechin equivalent. The extraction process was based on sonification. In another study by Rodriguez et al., extraction by sonification of Argentinian purple maize (flour) achieved values of 2.71 ± 0.04 mg GAE/g compared with those for white and yellow maize from India, with 1.6 and 1.3 mg GAE/g, respectively [56]. The concentration of polyphenols varies significantly among different genotypes of *Zea mays*.

**Figure 7:**
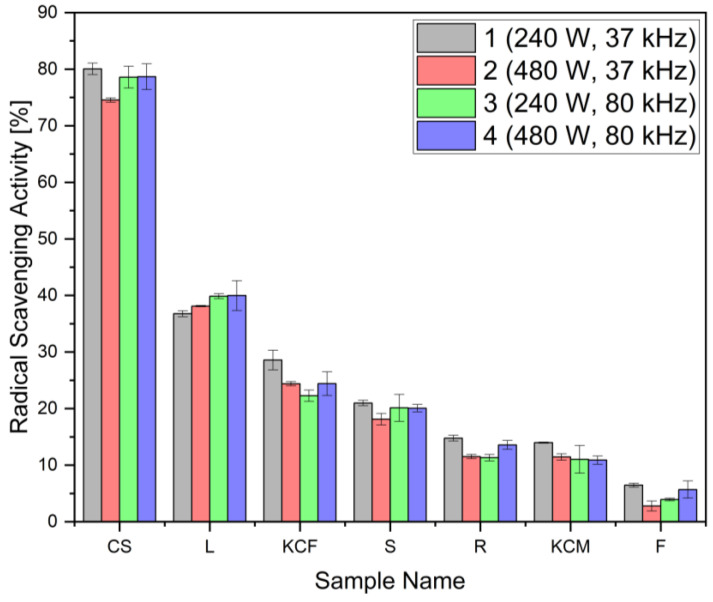
Radical scavenging activity of the seven maize parts.

The radical scavenging activity was assessed using a DPPH stable free radical. The results in Figure 7 indicate strong scavenging activity in all corn silk samples, with the highest observed for CS1 (80.06%), which corresponds to 9.34 mg of ascorbic acid equivalent per g of dry sample, while leaf samples exhibited approximately half of this activity (36.77% to 39.97%), ranking second. It is likely that the high concentration of polyphenols in corn silk is attributed to several functions within the plant. It may serve as the first line of defense against UV radiation and thus help mitigate oxidative stress induced in the kernels and the plant itself [57]. This could also explain why the leaves rank second, as they cover a large area to absorb UV light. Another possible reason for the high concentration of antioxidants in corn silk is its role in reproduction. The elevated levels of polyphenolic acid may contribute to the proper development of *Zea mays* [58]. The lowest radical scavenging activity was determined in the kernel samples (2.77% to 6.46%). All values for TPC and RSA can be seen in Table 2. In the radical scavenging activity assay, there were no statistical significance differences based on extraction parameters.

**Figure 8:**
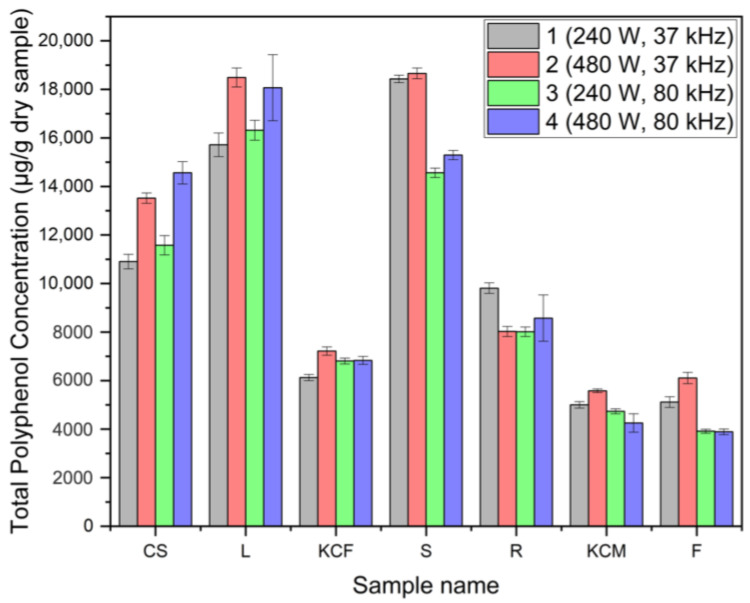
Total polyphenol concentration from HPLC of seven maize parts.

HPLC analysis confirmed the presence of up to 22 different polyphenolic compounds, which are provided in Table 3, along with their activity and the part of the plant with the highest concentration. *trans*-Cinnamic acid and kaempferol displayed the same retention time, making it unclear which substance was present. ANOVA was performed to identify any significant differences in extraction efficiency among the seven parts, based on the different parameters. In Figure 8, the total concentration of polyphenols in each part, as measured by HPLC, showed no statistical significance with respect to power, frequency, or their interaction. To further explore potential differences, statistical analysis was also conducted within each group (e.g., CS 1–4, F 1–4, etc.). These results were limited to the five most prevalent polyphenolic acids in each part of *Zea mays*, as they are expected to have the greatest impact on radical scavenging activity and total phenolic content. Figures S15–S21, showing the concentrations of all 22 polyphenolic acids, are included in the Supplementary Materials. In a comparison performed by Pérez-Jiménez et al. [20] regarding the top 100 richest dietary sources, our sample of corn silk would rank among the top 15 richest sources, based on the HPLC analysis, with 1090 to 1456 mg of polyphenols per 100 g. The sample of leaves would rank even higher, ranging from 1571 to 1848 mg per 100 g, and would end up ranking in the top 10.

**Figure 9:**
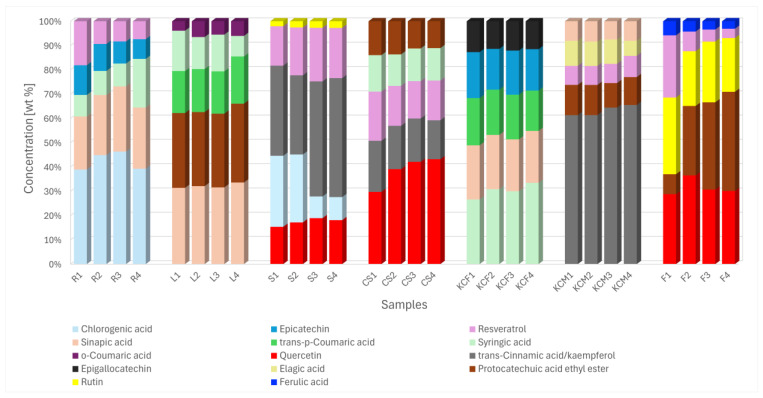
Content of five most frequent polyphenolic acids found in samples of *Zea mays*.

Figure 9 shows the five most frequent polyphenolic acids in each sample, divided into categories based on the part of the plant and the extraction parameter. There is no single predominant substance present in all the samples. Two-way repeated-measures ANOVA was performed for the samples in Figure 9 to determine any significant differences in the used power or frequency for extraction.

Corn silk (CS) extraction demonstrated a statistically significant effect of power on the extraction of quercetin and syringic acid. In contrast, root (R) samples exhibited various dependencies, with no significant differences observed for chlorogenic and syringic acid. Sinapic acid extraction was influenced by power, while epicatechin extraction was affected by frequency. Resveratrol was dependent on both power and frequency, but no interactions were found. Leaf (L) samples showed power dependency for sinapic acid and protocatechuic acid ethyl ester, while *trans*-*p*-coumaric and *o*-coumaric acid were influenced by both power and frequency, without interactions. Stem (S) samples exhibited similar dependencies, with *trans*-cinnamic acid and resveratrol affected by power, chlorogenic acid by frequency, and rutin showing no significant dependence. For the whole fermented plant (KCF), no correlations were found between power and frequency, except for in regards to syringic acid, which was power-dependent. Similarly, in the whole plant (KCM) samples, no statistical correlations were observed except for regarding the frequency of resveratrol extraction.

The most statistically significant differences were found in the kernel (F) samples. Quercetin extraction varied with frequency, particularly at 480 W. Protocatechuic acid ethyl ester was affected by interactions between power and frequency. Frequency had a significant effect, regardless of power, whereas power only influenced extraction at 37 kHz. Rutin extraction was significantly influenced by frequency, while power alone had no effect at either frequency. A significant interaction between power and frequency indicated that the effect of power depended on the frequency level. For ferulic acid, lower frequency resulted in a higher concentration, which was the only observed dependency. Resveratrol showed no statistical significance. The reason why the same polyphenols exhibit different behavior under various extraction parameters can be attributed to the varying structures of different plant parts and their distinct chemical compositions.

## 4. Conclusions

Quantitative and qualitative analyses of different parts of *Zea mays* revealed up to 22 different polyphenolic acids, all of which exhibit various activities and possible applications. All samples were tested using the radical scavenging activity assay, total phenolic content assay, ATR, HPLC, and UV–Vis spectroscopy. The bands in the ATR analysis confirm the presence of polyphenolic substances, according to the literature.

The sample with the greatest potential for therapeutic applications is corn silk. This sample exhibited the highest results for both radical scavenging activity and total phenolic content. No compound was identified that could be responsible for the observed high radical scavenging activity in the CS samples. However, the synergistic effect of the detected substances might be the cause. The leaves showed the second-highest activity, but their radical scavenging activity was almost half that of the corn silk.

It can be stated that the increase in radical scavenging activity occurs only with an overall increasing phenolic content. The different extraction parameters showed no significant influence on radical scavenging activity or total phenolic content, with slight changes in the root sample, where power and frequency were significant factors for achieving the highest concentration of phenolic acids.

According to the total phenolic content assay, the corn silk sample displayed a higher phenolic content than did the leaves but exhibited a lower overall content, according to the HPLC analysis. This is likely because the TPC assay can be influenced by other compounds. Another factor could be that not all polyphenolic acids display the same reactivity. These results suggest that potential bulk extraction processes can achieve the same yield with lower power, allowing for overall savings. Based on these results, it could be beneficial to conduct further research into the use of corn silk and leaves from corn plants for therapeutic purposes.

## Figures and Tables

**Table 1:** The parameters of the ultrasound used for extraction.

Number	Power (W)	Frequency (kHz)
**1**	240	37
2	480	37
3	240	80
4	480	80

**Table 2:** RSA values calculated per ascorbic acid (AAE) and gallic acid equivalents.

Sample	Radical Scavenging Activity (%)	mg AAE/g	mg GAE/g
CS1	80.06 ± 1.01	9.34 ± 0.0091	3.89 ± 0.0006
CS2	74.55 ± 0.38	8.70 ± 0.0034	4.07 ± 0.0004
CS3	78.58 ± 1.92	9.17 ± 0.0173	3.93 ± 0.0005
CS4	78.66 ± 2.27	9.18 ± 0.0204	3.88 ± 0.0005
R1	14.77 ± 0.50	1.73 ± 0.0045	2.21 ± 0.0006
R2	11.51 ± 0.37	1.35 ± 0.0033	2.07 ± 0.0010
R3	11.31 ± 0.58	1.33 ± 0.0052	1.92 ± 0.0004
R4	13.58 ± 0.77	1.59 ± 0.0069	1.78 ± 0.0006
F1	6.46 ± 0.35	0.76 ± 0.0031	0.75 ± 0.0002
F2	2.77 ± 0.90	0.34 ± 0.0081	0.91 ± 0.0001
F3	3.93 ± 0.23	0.47 ± 0.0021	0.77 ± 0.0002
F4	5.68 ± 1.52	0.67 ± 0.0137	0.69 ± 0.00008
L1	36.77 ± 0.53	4.30 ± 0.0048	3.16 ± 0.0003
L2	38.11 ± 0.11	4.45 ± 0.0010	3.09 ± 0.0003
L3	39.87 ± 0.43	4.66 ± 0.0038	3.31 ± 0.0001
L4	39.97 ± 2.62	4.67 ± 0.0236	3.17 ± 0.00006
KCM1	13.97 ± 0.11	1.64 ± 0.0010	1.92 ± 0.0008
KCM2	11.44 ± 0.58	1.35 ± 0.0052	1.60 ± 0.0002
KCM3	11.02 ± 2.44	1.30 ± 0.0220	1.60 ± 0.0006
KCM4	10.88 ± 0.72	1.28 ± 0.0064	1.39 ± 0.0001
S1	20.98 ± 0.47	2.46 ± 0.0043	2.82 ± 0.0002
S2	18.13 ± 1.01	2.12 ± 0.0091	2.70 ± 0.0006
S3	20.12 ± 2.38	2.36 ± 0.0214	2.77 ± 0.0010
S4	20.07 ± 0.64	2.35 ± 0.0058	2.75 ± 0.0006
KCF1	28.59 ± 1.72	3.34 ± 0.0155	3.03 ± 0.0003
KCF2	24.39 ± 0.36	2.86 ± 0.0033	2.97 ± 0.0005
KCF3	22.27 ± 0.99	2.61 ± 0.0089	2.94 ± 0.00008
KCF4	24.41 ± 2.10	2.86 ± 0.0189	2.90 ± 0.0010

**Table 3:** List of compounds detected by HPLC, with their activities, sources, and highest concentrations (*) observed in this study.

Name of Compound	Activity	Maize Part with Highest Concentration	Highest Concentration (µg/g)
Gallic acid	Antioxidant Anti-inflammatory Antitumor [59,60]	Leaves	35.53 ± 0.10
3,4-Dihydroxybenzoic (protocatechuic) acid	Antioxidant Anti-inflammatory Antibacterial Antitumor Antihyperlipidemic Antidiabetic Antiviral Neuroprotective [61,62,63]	KCF	286.33 ± 5.29
Neochlorogenic acid	Antioxidant Antifungal Anti-inflammatory Antitumor [64,65,66]	Stem	125.5 ± 3.60
4-Hydroxybenzoic acid	Antioxidant Antimicrobial Antifungal Anti-inflammatory Antimutagenic Antisickling Antidiabetic Anticancer [67,68]	Corn silk	8.94 ± 0.05
Epigallocatechin	Antioxidant Antimutagenic Antitumor Antiplatelet Anticoagulation [69,70]	KCF	545.44 ± 8.98
Catechin	Antioxidant Anti-inflammatory Antidiabetic Antitumor [71,72,73]	KCF	553.30 ± 7.27
Vanillic acid	Antioxidant Anti-inflammatory Anti-venom Antimicrobial [74,75,76]	KCF	174.69 ± 9.75
Chlorogenic acid	Antioxidant Antibacterial Anti-inflammatory Anticarcinogenic [77,78]	Root	2828.28 ± 56.32
Caffeic acid	Antioxidant Anti-inflammatory Anti-anxiety Anti-depressive [79,80,81]	KCF	459.49 ± 7.05
Syringic acid	Antioxidant Anti-inflammatory Anticancer Antinociceptive Antimicrobial [82,83]	Leaves	2117.48 ± 257.54
Epicatechin	Antioxidant Anti-inflammatory [84,85]	KCF	805.48 ± 6.52
*trans*-*p*-Coumaric acid	Antioxidant Anti-inflammatory [86,87]	Leaves	2952.06 ± 5.93
Ferulic acid	Antioxidant Anti-inflammatory Anticarcinogenic Antimicrobial Antiviral Antidiabetic [88,89]	Leaves	510.129 ± 26.90
Sinapic acid	Antioxidant Anti-inflammatory Anticancer Antimicrobial Antidiabetic [90,91]	Leaves	5100.68 ± 80.76
Ellagic acid	Antioxidant Anticancer Antibacterial [92,93]	Corn silk	399.91 ± 0.37
Rutin	Antioxidant Anticancer Antidiabetic [94,95]	Kernel	1253.04 ± 1.91
*trans*-2-Hydroxycinnamic (*o*-coumaric) acid	Antioxidant Anticarcinogenic [96,97]	Corn silk	1462.91 ± 1.36
Protocatechuic acid ethyl ester	Antioxidant Antimicrobial Antitumor Anti-inflammatory [98,99]	Leaves	4927.28 ± 97.37
Resveratrol	Antioxidant Anti-inflammatory Anticancer Cardioprotective Neuroprotective [100,101]	Stem	3175.9 ± 49.40
*trans*-Cinnamic acid *	Antioxidant Anti-inflammatory Antimicrobial [102,103]	Stem	6234.6 ± 19.60
Kaempferol *	Antioxidant Anti-inflammatory Antibacterial Anticarcinogenic Antidiabetic [104,105]	Stem	6234.6 ± 19.60
Quercetin	Antioxidant Anti-inflammatory Cardioprotective Anticancer Antiviral [106,107]	Corn silk	4820.44 ± 141.74

## Data Availability

The original contributions presented in the study are included in the article, further inquiries can be directed to the corresponding author.

## Supplementary Materials

The following supporting information can be downloaded at https://www.mdpi.com/article/10.3390/foods14091458/s1: Figure S1: Bar graph of 22 polyphenolic acids and their concentration in whole fermented corn determined by HPLC; Figure S2: Bar graph of 22 polyphenolic acids and their concentration in whole corn determined by HPLC; Figure S3: Bar graph of 22 polyphenolic acids and their concentration in fruit determined by HPLC; Figure S4: Bar graph of 22 polyphenolic acids and their concentration in leaves determined by HPLC; Figure S5: Bar graph of 22 polyphenolic acids and their concentration in stem determined by HPLC; Figure S6: Bar graph of 22 polyphenolic acids and their concentration in root determined by HPLC; Figure S7: Bar graph of 22 polyphenolic acids and their concentration in Corn silk determined by HPLC; Figure S8: ATR spectra of CS1-4 samples; Figure S9: ATR spectra of F1-4 samples; Figure S10: ATR spectra of KCF1-4 samples; Figure S11: ATR spectra of KCM1-4 samples; Figure S12: ATR spectra of L1-4 samples; Figure S13: ATR spectra of R1-4 samples; Figure S14: ATR spectra of S1-4 samples; Figure S15: UV/VIS spectra of KCF1-4 samples; Figure S16: UV/VIS spectra of F1-4 samples; Figure S17: UV/VIS spectra of CS1-4 samples; Figure S18: UV/VIS spectra of KCM1-4 samples; Figure S19: UV/VIS spectra of L1-4 samples; Figure S20: UV/VIS spectra of R1-4 samples; Figure S21: UV/VIS spectra of S1-4 samples.

## Abbreviations

The following abbreviations, listed in alphabetical order, are used in this manuscript:

AAE	ascorbic acid equivalent
CS	corn silk
DPPH	2,2-Diphenyl-1-picrylhydrazyl
F	kernel
FCR	Folin–Ciocalteu reagent
GAE	gallic acid equivalent
KCF	whole fermented plant
KCM	whole plant
L	leaf
R	root
ROS	reactive oxygen species
RSA	radical scavenging activity
S	stem
TPC	total phenolic content
UAE	ultrasound-assisted extraction
