# Supplementary material for: Comparative Analysis of Polyphenolic Acids from Various Zea mays Parts in Ultrasound-Assisted Extraction
Source: Foods. 2025 Apr 23;14(9):1458. doi: 10.3390/foods14091458 (PMC12071472; doi:10.3390/foods14091458)
Supplement: Supplementary file 1 [file foods-14-01458-s001.zip › foods-3562059-supplementary.pdf]

Comparative Analysis of Polyphenolic Acids from Various *Zea mays* Parts in Ultrasound-Assisted Extraction

David Řepka [1], Lubomír Lapčík [1,2,*]

[1] Department of Physical Chemistry, Faculty of Science, Palacky University, 17. Listopadu 12, 771 46 Olomouc, Czech Republic; david.repka@upol.cz
[2] Department of Foodstuff Technology, Faculty of Technology, Tomas Bata University in Zlin, Nam. T.G. Masaryka 275, 762 72 Zlin, Czech Republic
* Correspondence: lapcikl@seznam.cz

HPLC

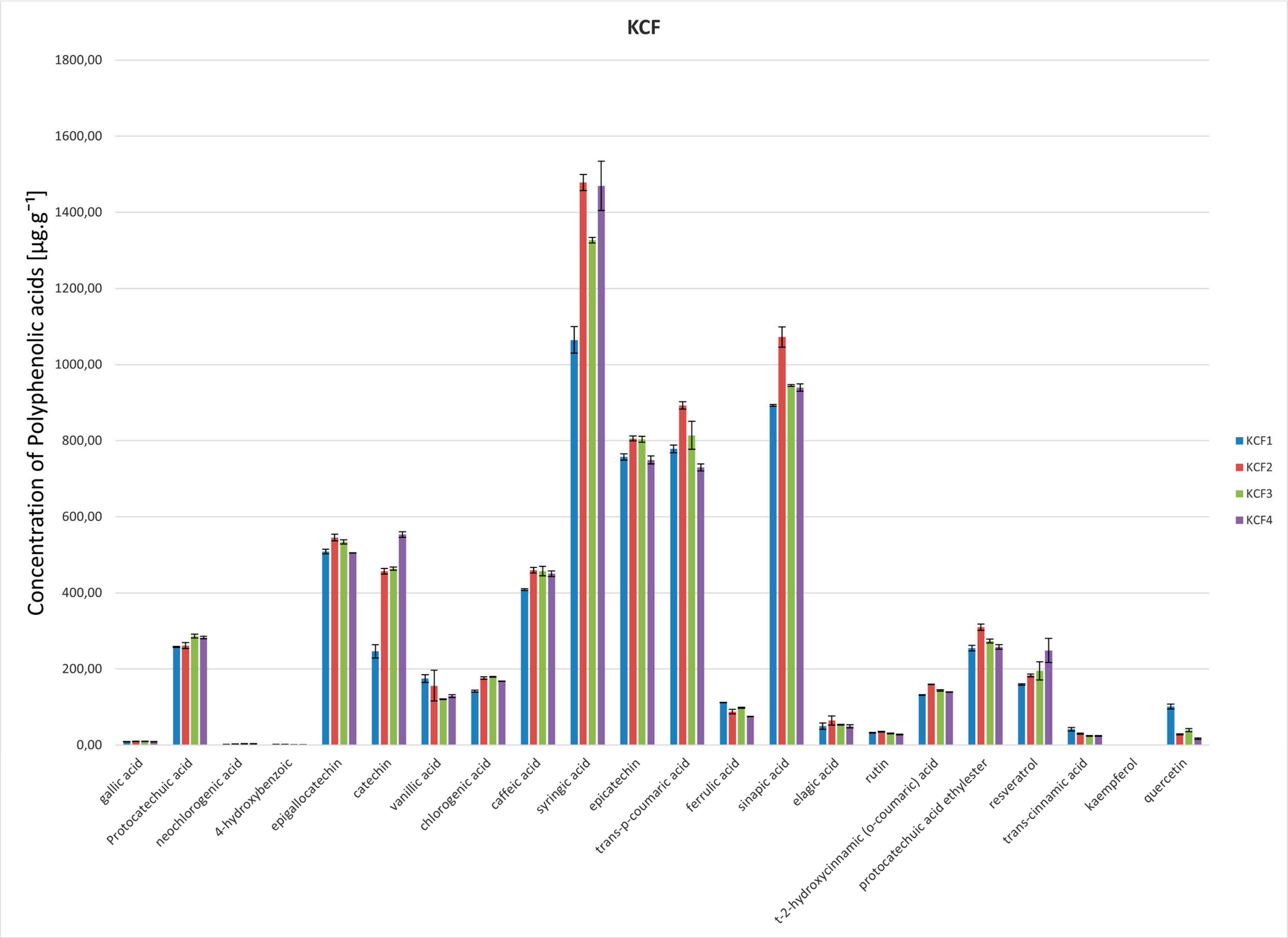

Figure S1: Bar graph of 22 polyphenolic acids and their concentration in whole fermented corn determined by HPLC

HPLC

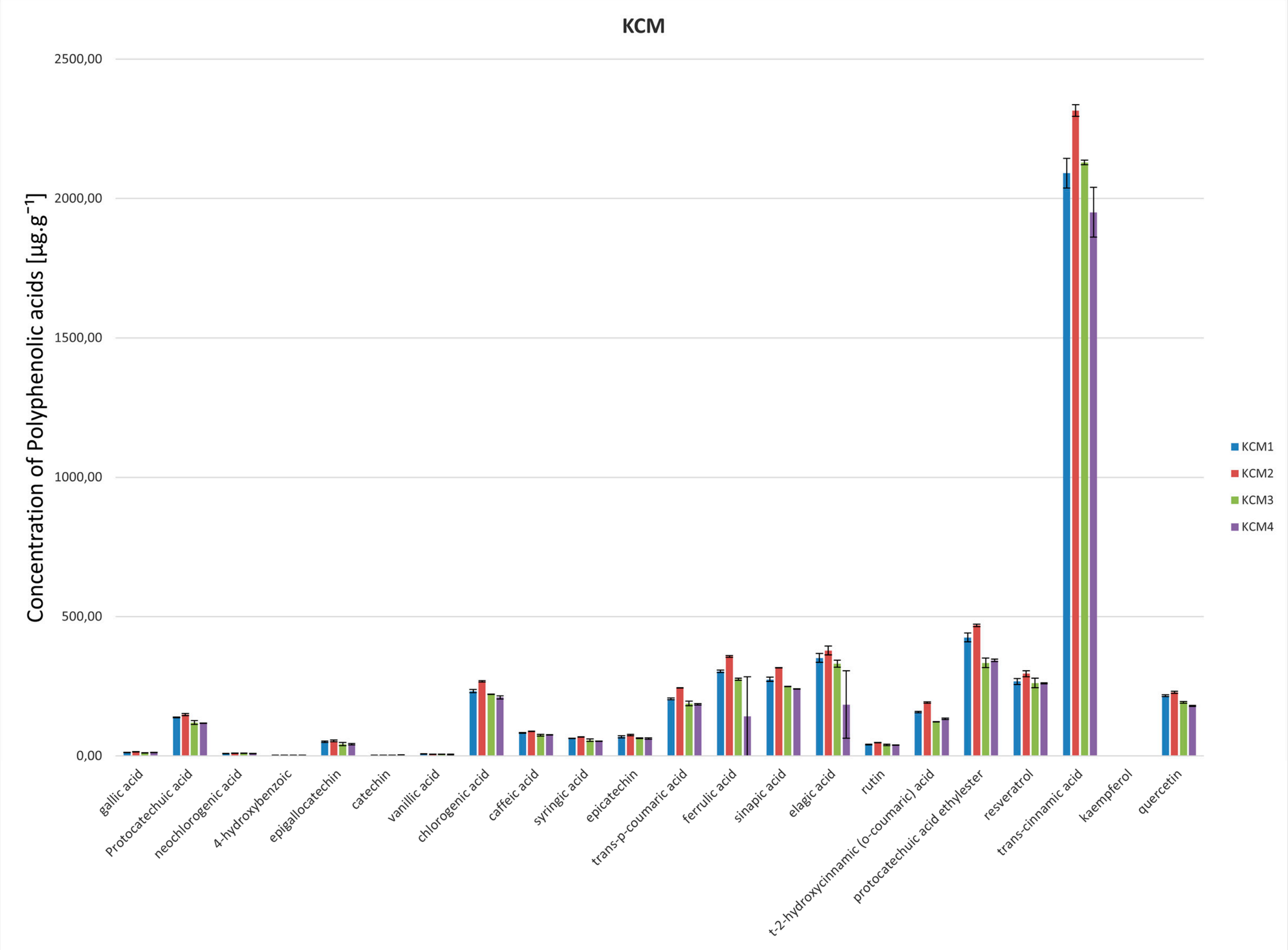

Figure S2: Bar graph of 22 polyphenolic acids and their concentration in whole corn determined by HPLC

HPLC

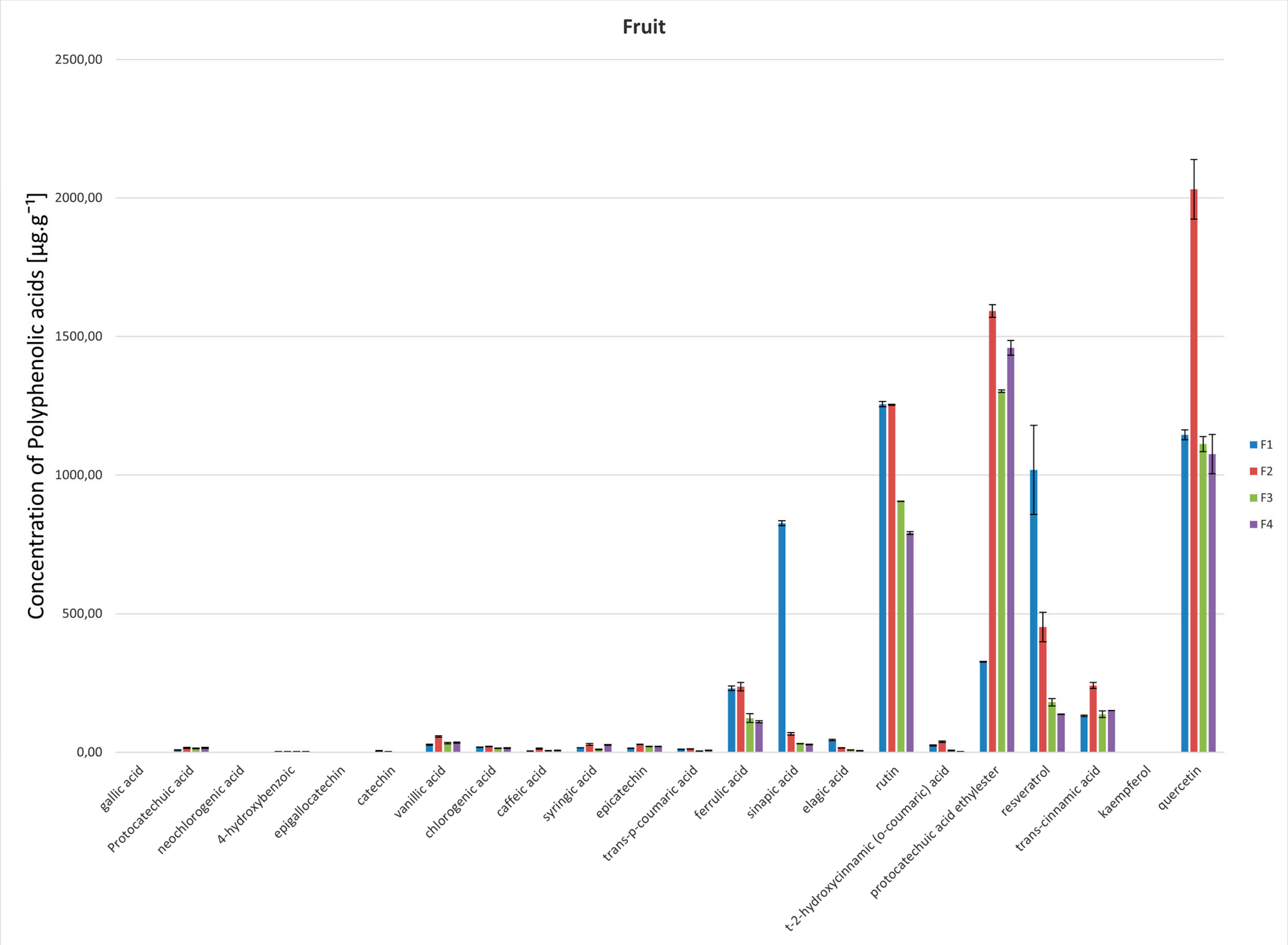

Figure S3: Bar graph of 22 polyphenolic acids and their concentration in fruit determined by HPLC

HPLC

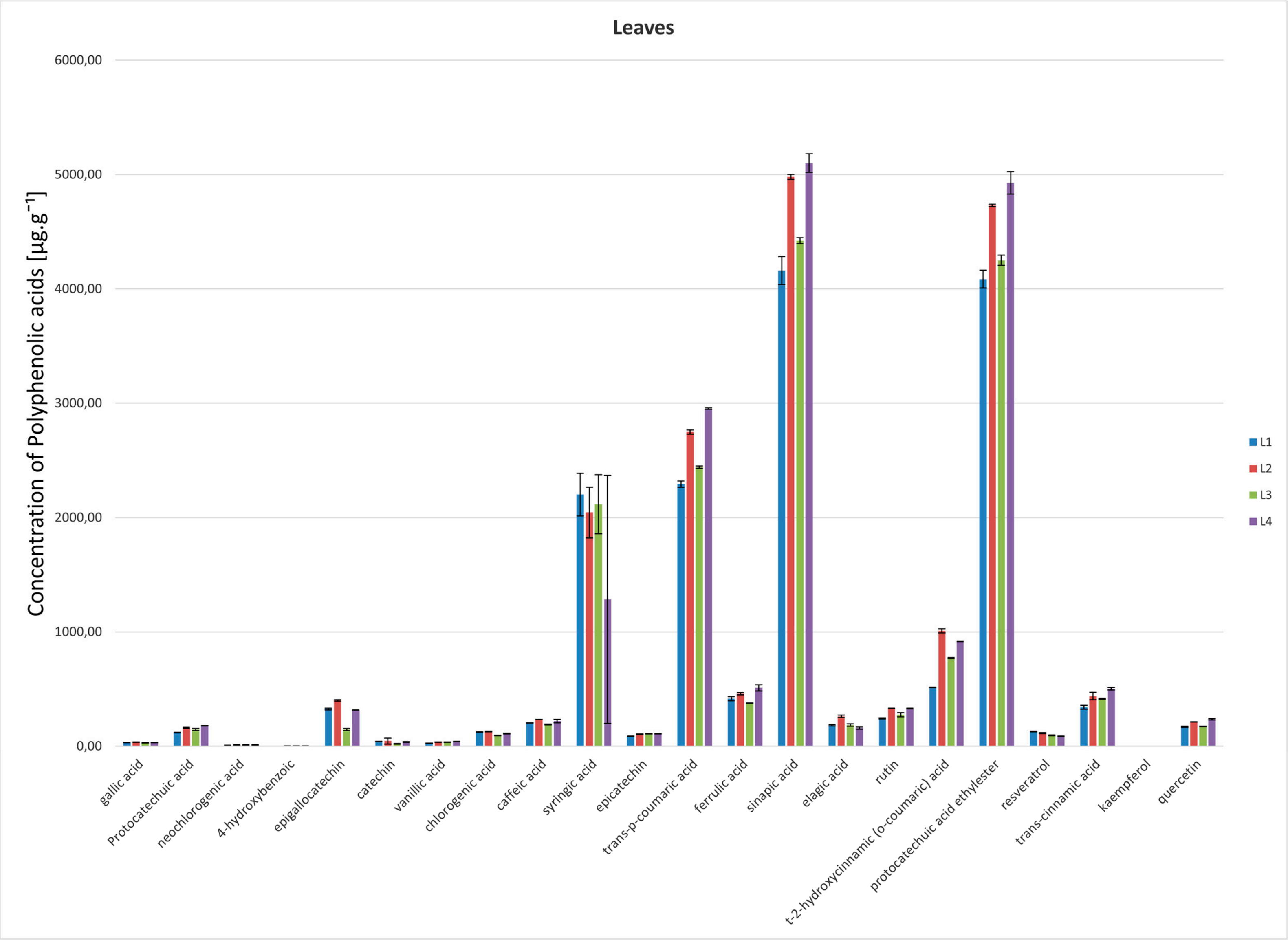

Figure S4: Bar graph of 22 polyphenolic acids and their concentration in leaves determined by HPLC

HPLC

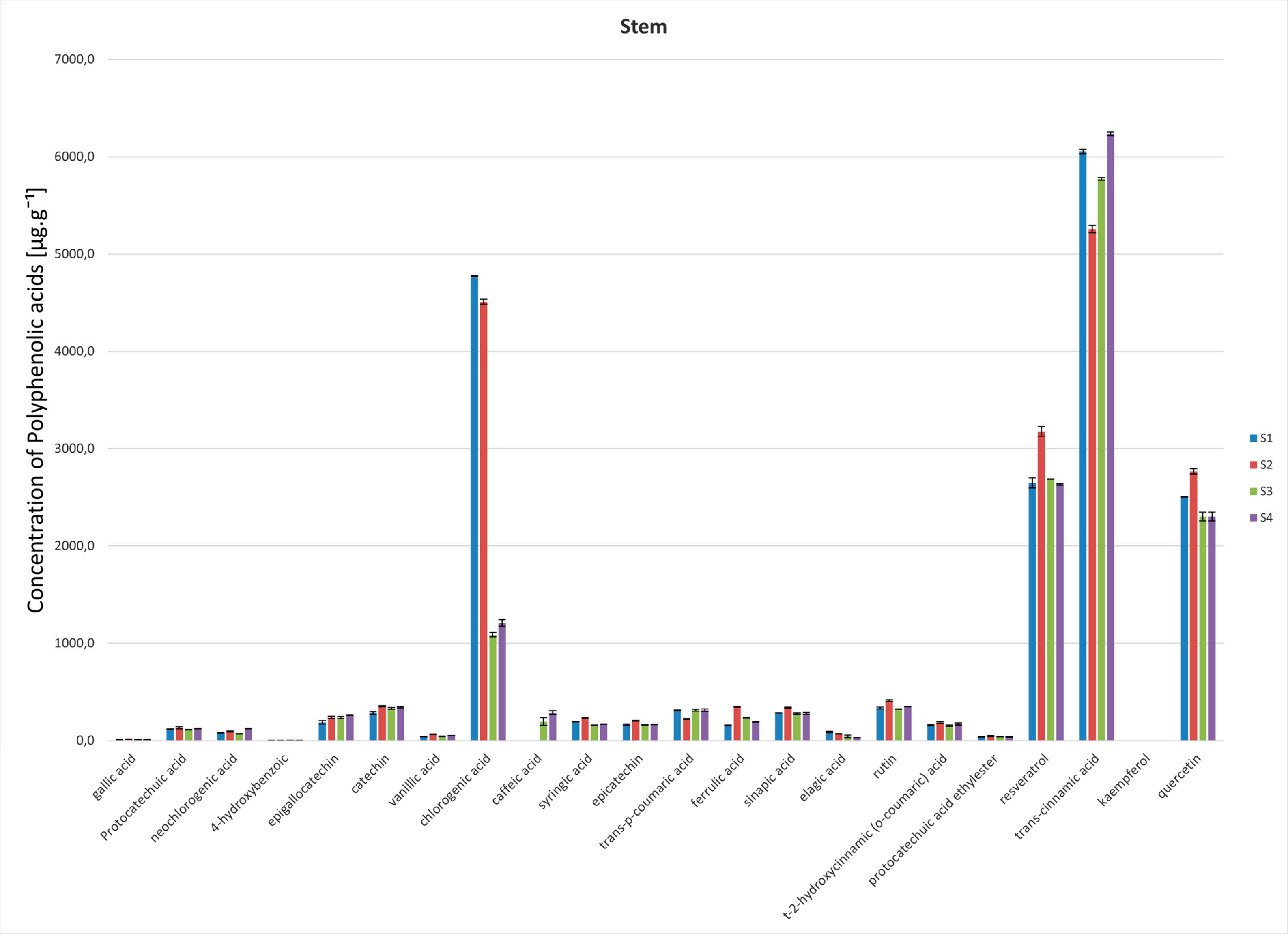

Figure S5: Bar graph of 22 polyphenolic acids and their concentration in stem determined by HPLC

HPLC

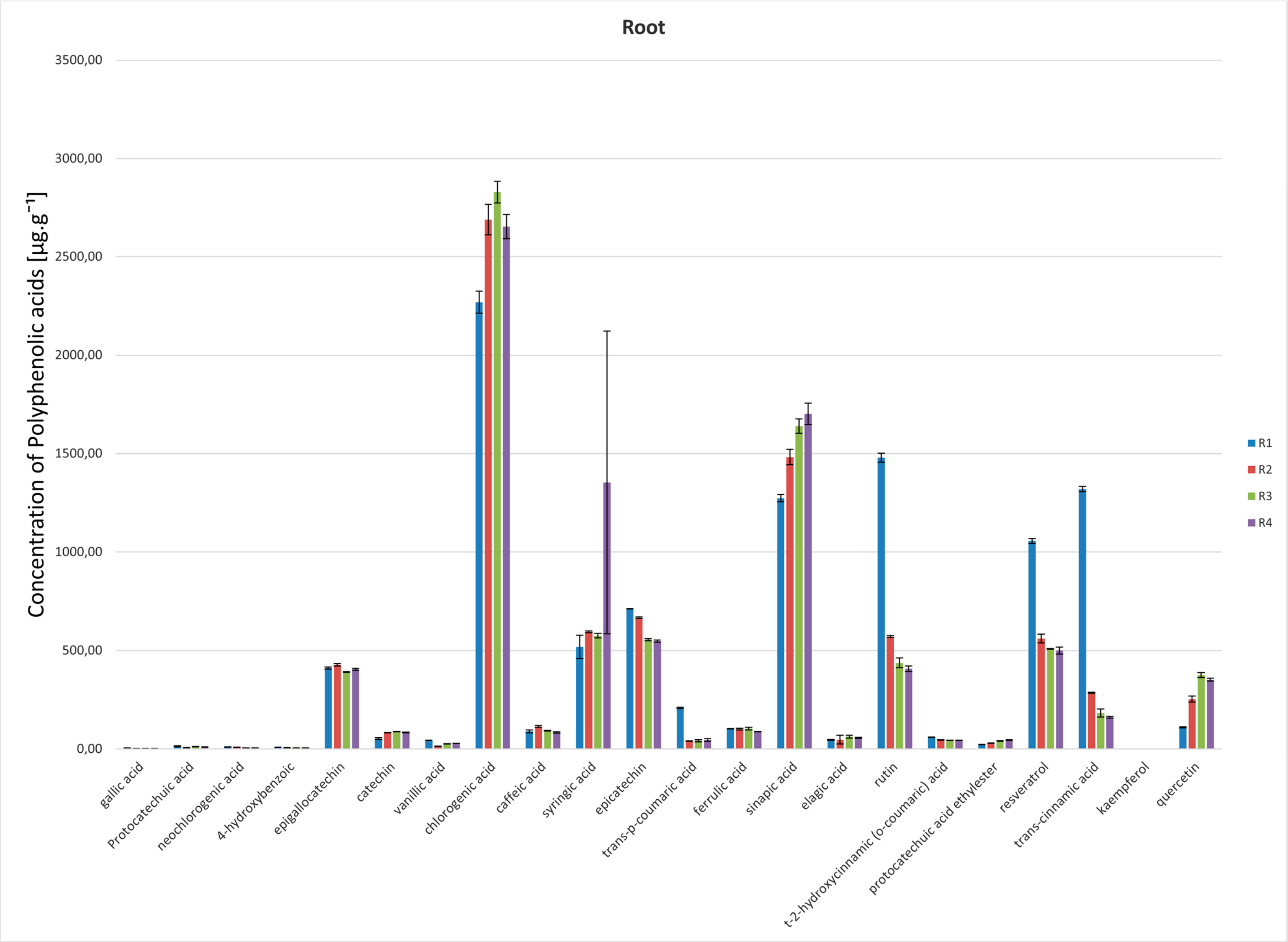

Figure S6: Bar graph of 22 polyphenolic acids and their concentration in root determined by HPLC

HPLC

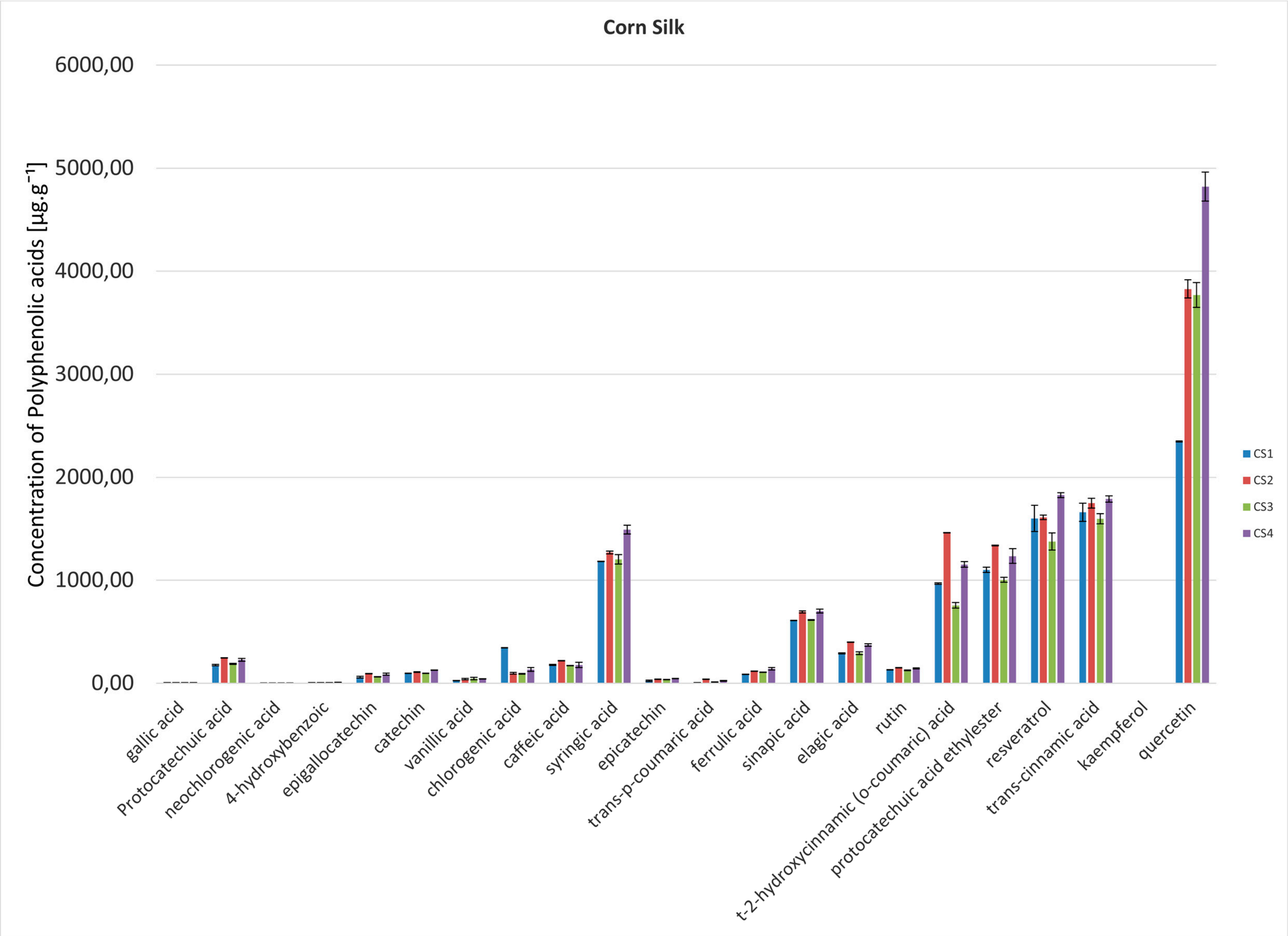

Figure S7: Bar graph of 22 polyphenolic acids and their concentration in Corn silk determined by HPLC

UV/VIS

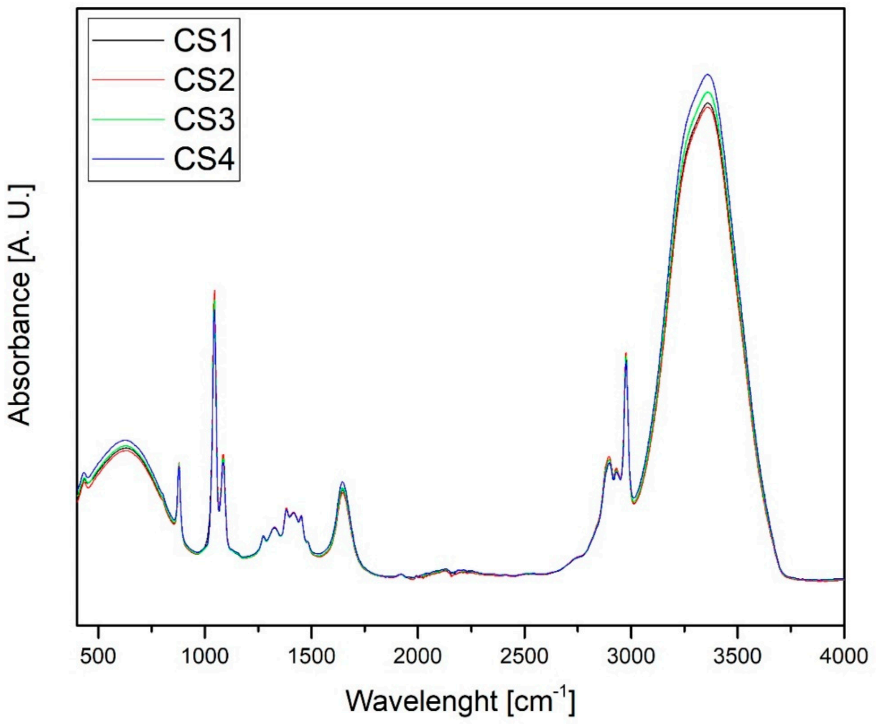

Figure S8: ATR spectra of CS1-4 samples

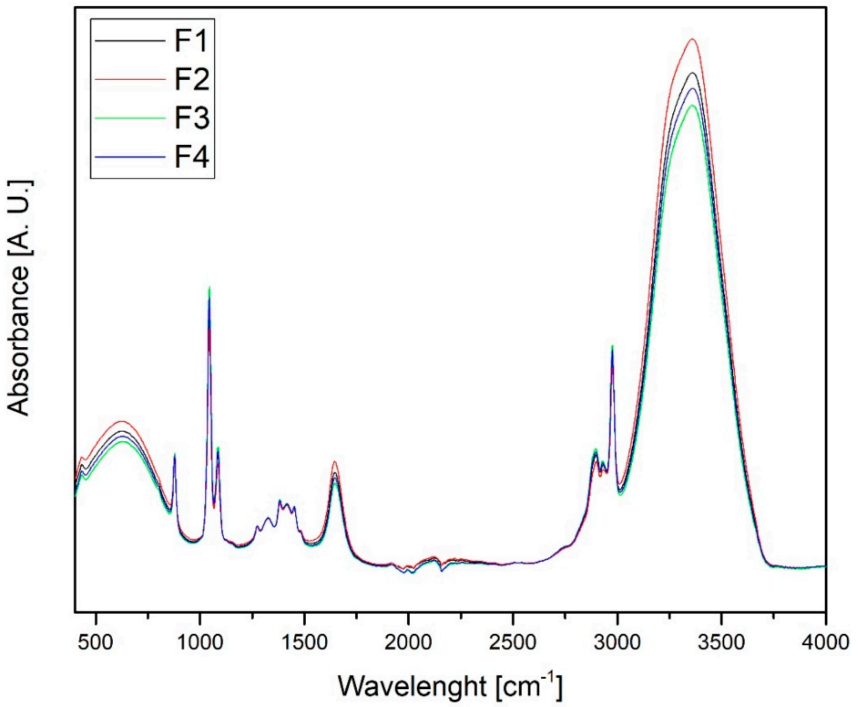

Figure S9: ATR spectra of F1-4 samples

UV/VIS

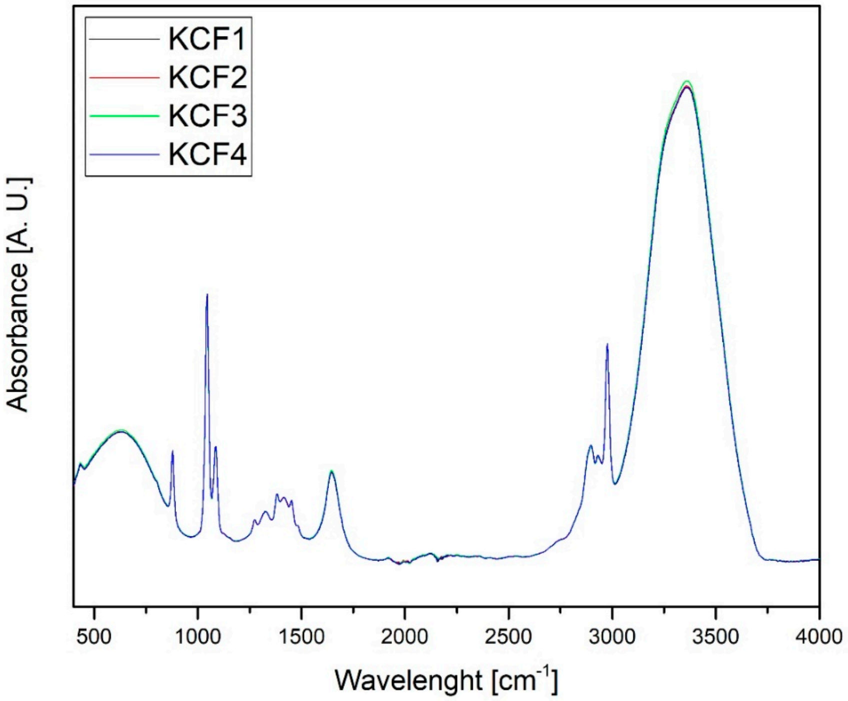

Figure S10: ATR spectra of KCF1-4 samples

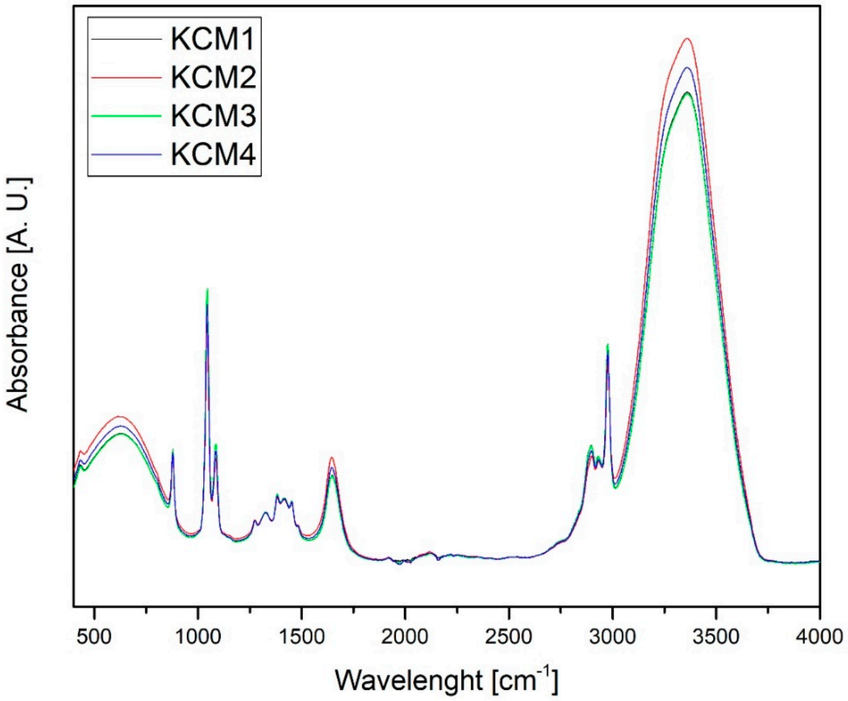

Figure S11: ATR spectra of KCM1-4 samples

UV/VIS

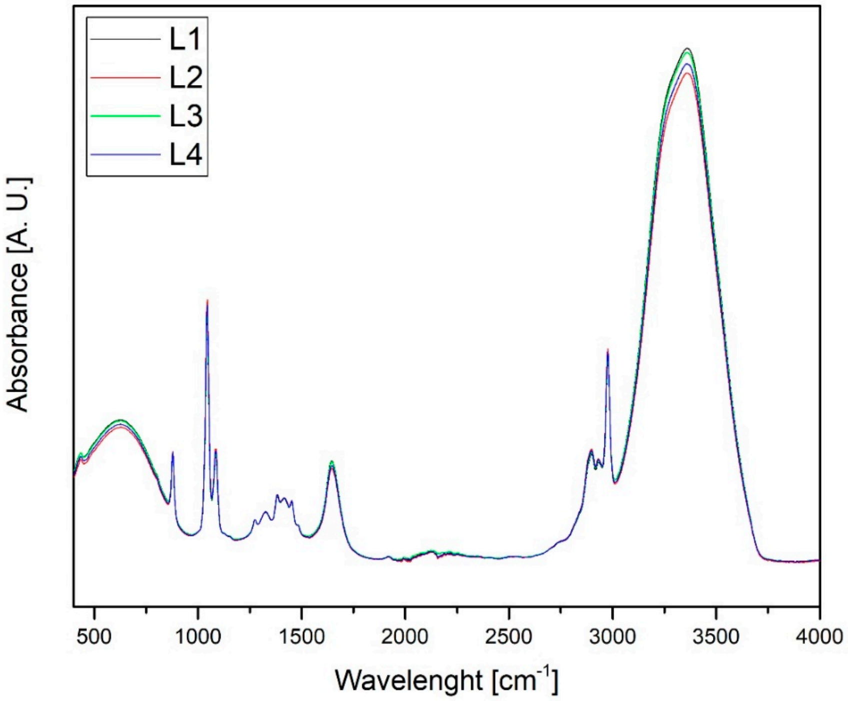

Figure S12: ATR spectra of L1-4 samples

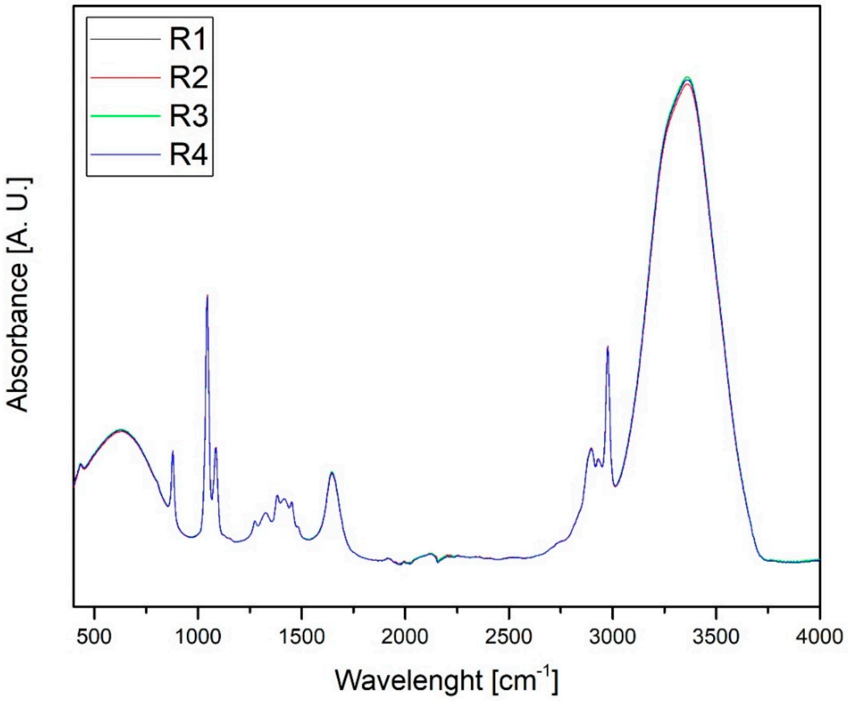

Figure S13: ATR spectra of R1-4 samples

UV/VIS

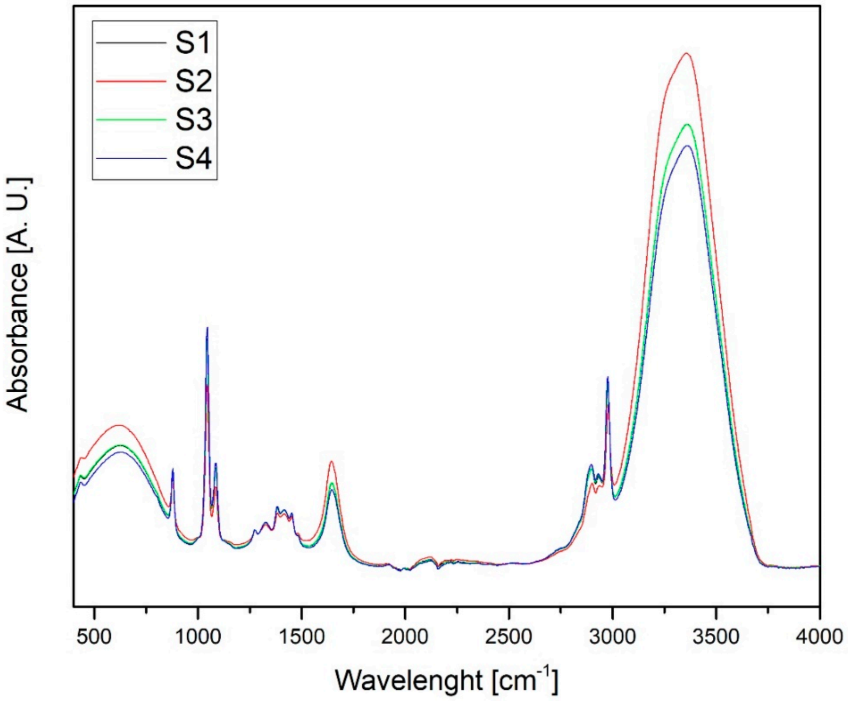

Figure S14: ATR spectra of S1-4 samples

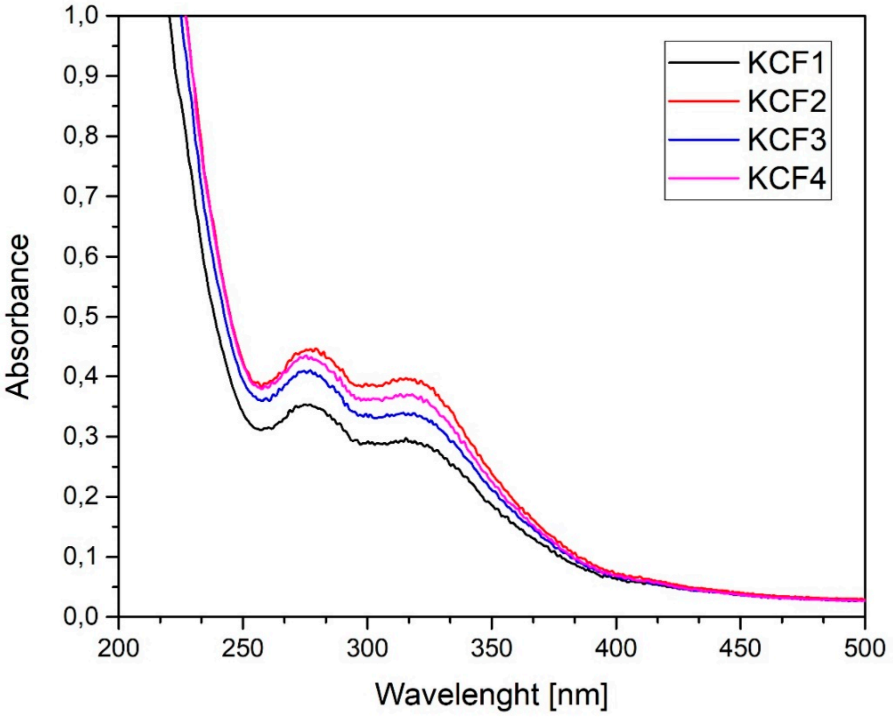

Figure S15: UV/VIS spectra of KCF1-4 samples

UV/VIS

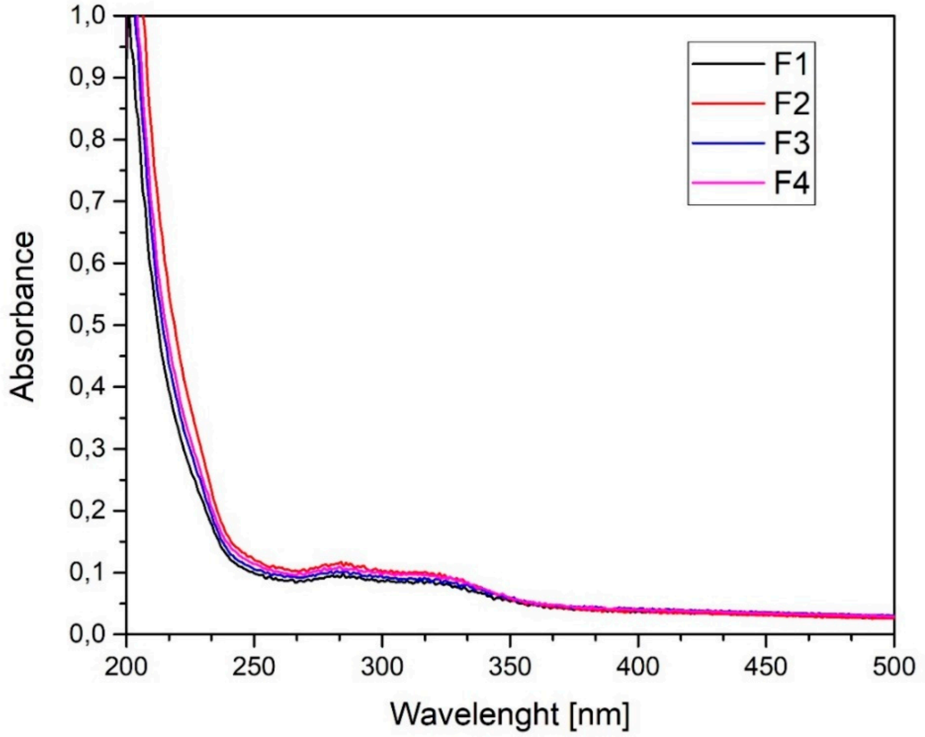

Figure S16: UV/VIS spectra of F1-4 samples

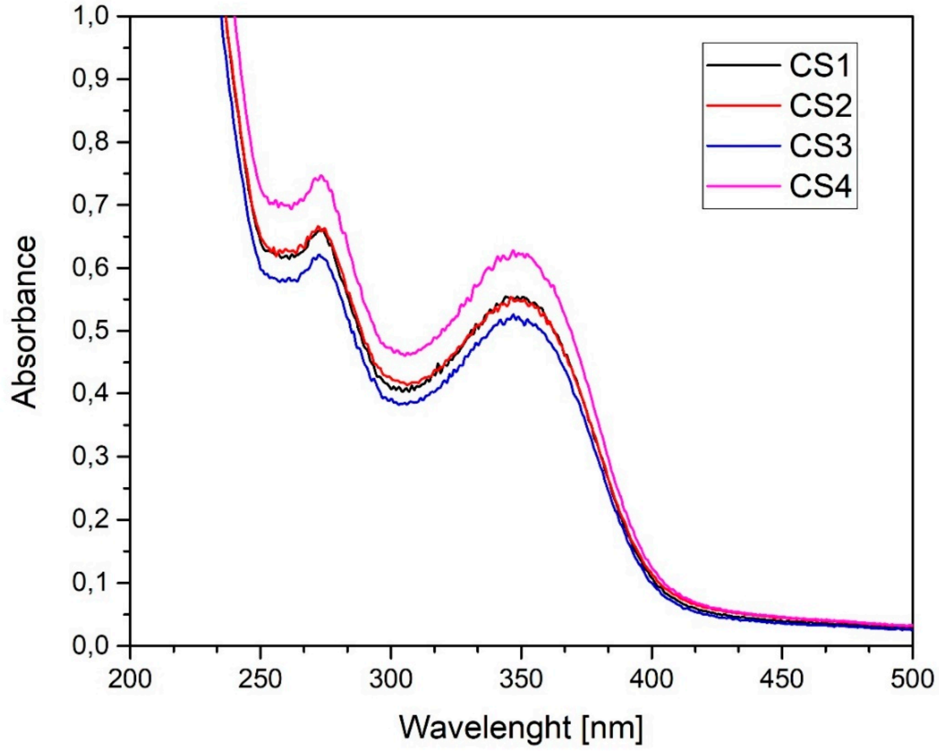

Figure S17: UV/VIS spectra of CS1-4 samples

UV/VIS

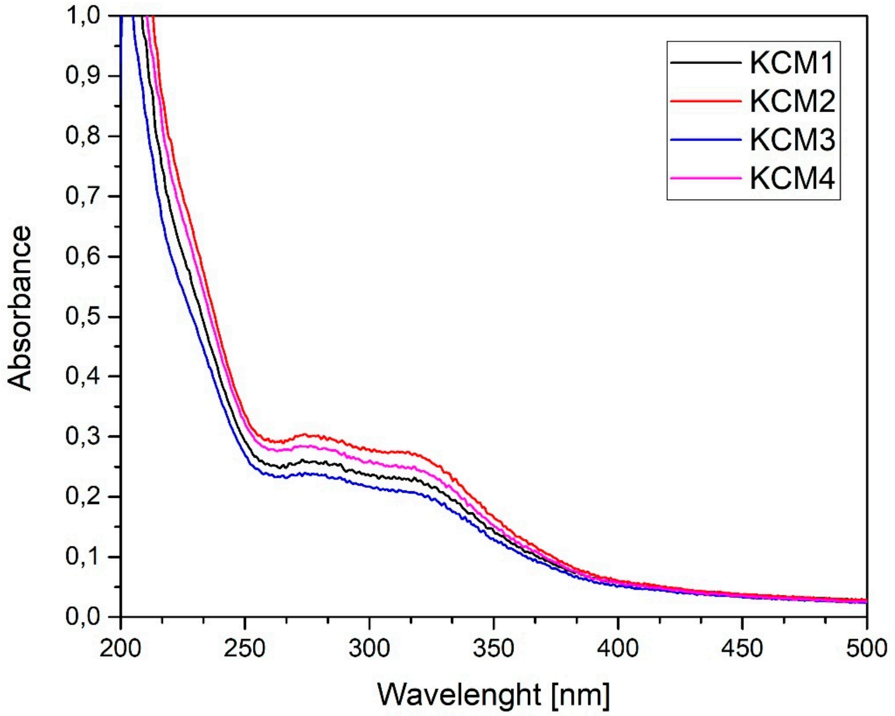

Figure S18: UV/VIS spectra of KCM1-4 samples

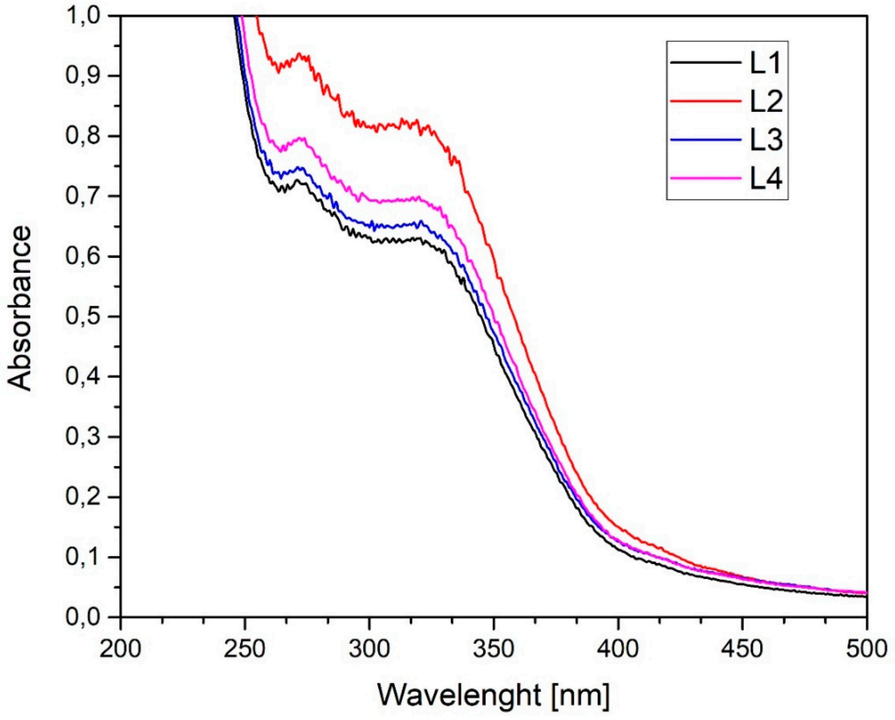

Figure S19: UV/VIS spectra of L1-4 samples

UV/VIS

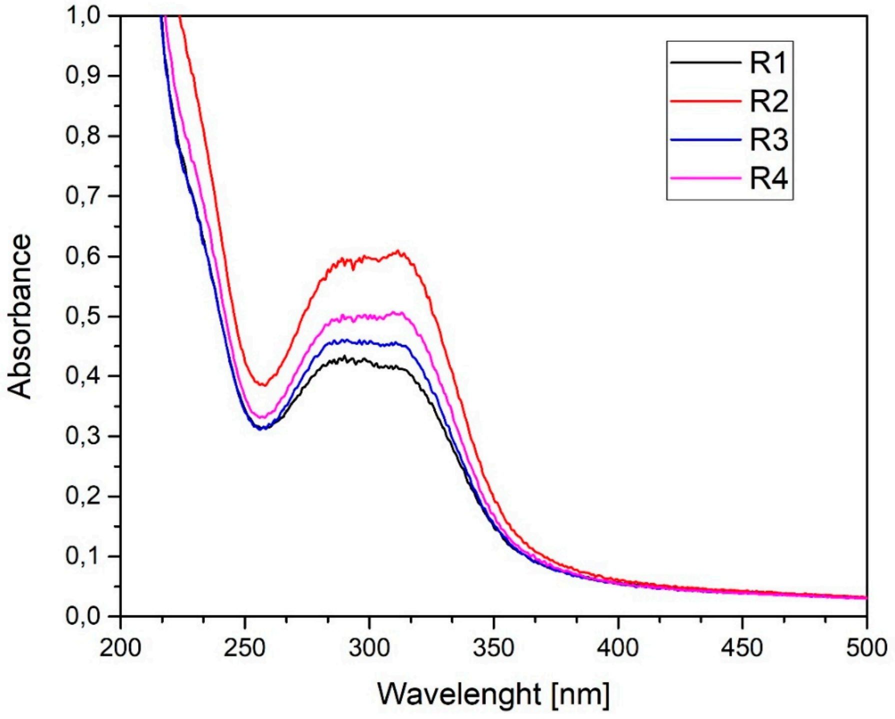

Figure S20: UV/VIS spectra of R1-4 samples

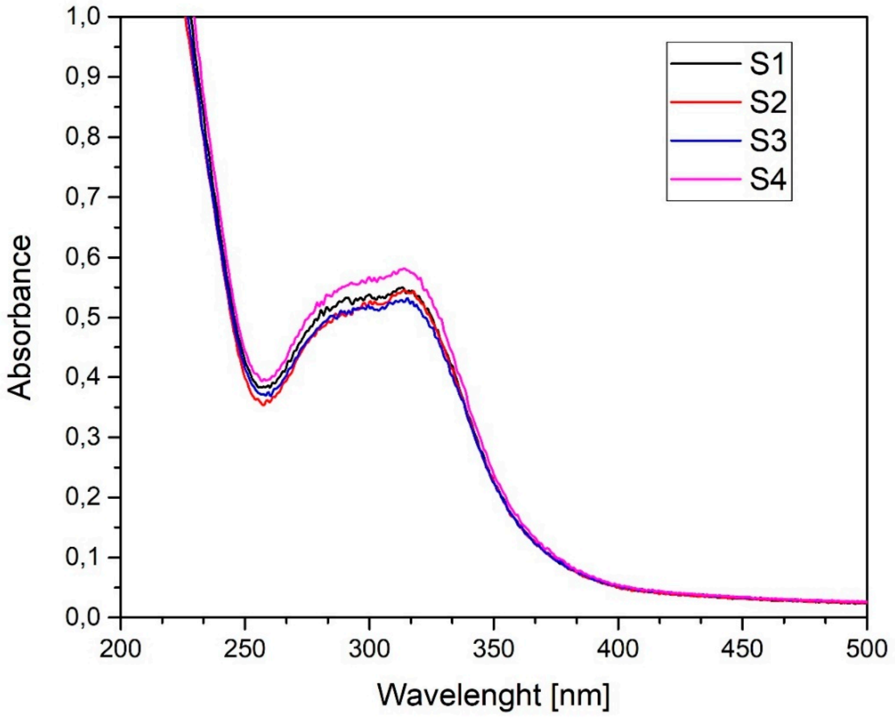

Figure S21: UV/VIS spectra of S1-4 samples
